# Mapping of Structure-Function Age-Related Connectivity Changes on Cognition Using Multimodal MRI

**DOI:** 10.3389/fnagi.2022.757861

**Published:** 2022-05-18

**Authors:** Daiana Roxana Pur, Maria Giulia Preti, Anik de Ribaupierre, Dimitri Van De Ville, Roy Eagleson, Nathalie Mella, Sandrine de Ribaupierre

**Affiliations:** ^1^Schulich School of Medicine & Dentistry, Western University, London, ON, Canada; ^2^CIBM Center for Biomedical Imaging, Lausanne, Switzerland; ^3^Institute of Bioengineering, Center for Neuroprosthetics, EPFL, Geneva, Switzerland; ^4^Department of Radiology and Medical Informatics, University of Geneva (UNIGE), Geneva, Switzerland; ^5^Department of Psychology, University of Geneva, Geneva, Switzerland; ^6^Department of Electrical and Computer Engineering, Western University, London, ON, Canada; ^7^The Brain and Mind Institute, Western University, London, ON, Canada; ^8^Department of Clinical Neurological Sciences, Schulich School of Medicine, Western University, London, ON, Canada

**Keywords:** fMRI, functional connectivity, structural connectivity, healthy aging, variability, neuroimaging, structure-function coupling

## Abstract

The relationship between age-related changes in brain structural connectivity (SC) and functional connectivity (FC) with cognition is not well understood. Furthermore, it is not clear whether cognition is represented *via* a similar spatial pattern of FC and SC or instead is mapped by distinct sets of distributed connectivity patterns. To this end, we used a longitudinal, within-subject, multimodal approach aiming to combine brain data from diffusion-weighted MRI (DW-MRI), and functional MRI (fMRI) with behavioral evaluation, to better understand how changes in FC and SC correlate with changes in cognition in a sample of older adults. FC and SC measures were derived from the multimodal scans acquired at two time points. Change in FC and SC was correlated with 13 behavioral measures of cognitive function using Partial Least Squares Correlation (PLSC). Two of the measures indicate an age-related change in cognition and the rest indicate baseline cognitive performance. FC and SC—cognition correlations were expressed across several cognitive measures, and numerous structural and functional cortical connections, mainly cingulo-opercular, dorsolateral prefrontal, somatosensory and motor, and temporo-parieto-occipital, contributed both positively and negatively to the brain-behavior relationship. Whole-brain FC and SC captured distinct and independent connections related to the cognitive measures. Overall, we examined age-related function-structure associations of the brain in a comprehensive and integrated manner, using a multimodal approach. We pointed out the behavioral relevance of age-related changes in FC and SC. Taken together, our results highlight that the heterogeneity in distributed FC and SC connectivity patterns provide unique information about the variable nature of healthy cognitive aging.

## Introduction

Aging is associated with heterogenous changes in cognition and in the structure and function of the brain (Kennedy and Raz, 2015; Damoiseaux, 2017). Research over the past decades has yet to elucidate the effect of age on the association between functional and structural brain networks. Various “disruptive” or “disconnection” theories suggest that cognitive decline in normal aging stems from alterations in the integration of functional properties of brain networks and/or from subtle anatomical disconnection between brain regions, possible due to microstructural white matter loss or demyelination (O’Sullivan et al., 2001; Salat et al., 2005; Andrews-Hanna et al., 2007).

Longitudinal studies of aging indicate that changes in cognition vary considerably across different cognitive domains and behavioral tasks (i.e., visuo-spatial abilities, reaction time, processing speed, planning, decision making) at the inter-individual and even intra-individual level (Kliegel and Sliwinski, 2004; Salthouse, 2009; Goh et al., 2013; Mella et al., 2015). For example, some individuals may experience a general cognitive decline across multiple behavioral tasks (i.e., homogenous decline), while others may decline in one cognitive ability but experience preservation or even improvement of others (i.e., heterogenous change). A hallmark of cognitive aging is decreased processing speed, or an inferior performance on perceptual, motor, and decision-making tasks (Salthouse, 2000). Processing speed involves coordinated activity across multiple neural networks, and so engages perception, decision making, planning, motor performance, and task evaluation (Salthouse, 1996; Eckert et al., 2010). Therefore, processing speed tasks could be evaluating any of these abilities.

The small number of longitudinal studies that investigated changes in functional connectivity (FC) in healthy older adults were inconsistent, some indicate stability (Persson et al., 2014), while others reported a decline in intra-network FC in the executive control network and default mode network (DMN) as well as an initial increase in inter-network FC between the executive control network and DMN followed by a subsequent decline with older age (Ng et al., 2016) On the other hand, some studies suggest a pattern of initial decrease and subsequent temporary increase in FC (Cao et al., 2014; Damoiseaux, 2017). These results are generally attributed to the variability of compensatory or over-recruitment mechanisms across different cognitive domains (reviewed by Betzel et al., 2014; Damoiseaux, 2017). For example, the initial increase in FC can be attributed to an attempt to compensate for declining function, a strategy that cannot be sustained over time. Studies on structural changes with aging are more consistent, with most results pointing to widespread decreases in fractional anisotropy, particularly in frontal brain regions (Betzel et al., 2014; Zhao et al., 2015; Damoiseaux, 2017).

Most studies on the topic of FC and SC changes in aging are cross-sectional, focusing on group mean differences rather than longitudinal individual-level age-related changes (i.e., the aging process itself; Damoiseaux, 2017). Zimmermann et al. (2016) examined the effect of age on the correlation of FC and structural connectivity (SC) in a small, adult lifespan sample, and found that age-related changes in FC and SC coupling are region-dependent.

The current study examined the effect of aging on the change in the structural and functional integrity of the brain while also considering individual differences in cognition. Specifically, the main objective of the present study was to investigate how changes in FC and SC derived from magnetic resonance imaging (MRI), over a span of 2 and a half years, correlate with changes in different cognitive measures using a multivariate framework. Partial least squares (PLS) were used to map orthogonal patterns of brain-cognition relationships (McIntosh and Lobaugh, 2004; Krishnan et al., 2011).

Overall, we expected FC and SC—cognition correlations to be captured by variations in the pattern and amplitude of change across various cognitive measures (i.e., heterogeneity and amplitude of change). Additionally, it was expected that the brain-behavior relationship was positively and negatively associated with numerous structural and functional connections, mainly relating to regions susceptible to age-related change such as cingulo-opercular, dorsolateral prefrontal, somatosensory and motor, and temporo-parieto-occipital. One of the novel aspects of the current study is the use of the measure of change in brain connectivity as well as of behavioral change, which should better reflect changes related to the aging process rather than just age-related differences.

## Materials and Methods

### Participants

The participants of the current study came from the longitudinal Geneva Aging Study. They all provided written informed consent, and approval was obtained from the ethics committee of the Faculty of Psychology and Educational Sciences of the University of Geneva and the Swiss Ethics Committee.

Older subjects that underwent two T1-weighted structural images, diffusion-weighted images, and fMRI scans, as well as a battery of cognitive tests were selected from a larger pool of the Geneva Aging Study, a lifespan study of 219 older adults (Fagot et al., 2018). The current analyses focused on a subset of individuals that completed longitudinal follow-up at ~2.5 years including behavioral testing and scanning. Subjects missing any one of the behavioral tests or scans were not included. At this stage, 31 subjects were selected. Two subjects were excluded due to additional missing behavioral data and one because of a lesion on the anatomical MRI. The final sample included 28 older subjects (mean age at first scan = 72 ± 6 years, mean age at second scan = 74 ± 6 years; eight males). Their demographic information is presented in Table 1. Participants were screened for health problems with a health questionnaire and their structural MRIs were inspected by a neurosurgeon with significant experience evaluating MRIs (SR) to rule out abnormalities. The battery of the behavioral tests was systematically administered before the scanning sessions (mean interval between testing and MRI = 57 days, SD = 39). Image quality was additionally ensured by examining head motion: absolute head motion at a single time point >2 mm and relative head motion >2.5 mm were criteria for participant removal. No participants were removed.

**Table 1:** Participant demographic information.

Demographic	
Age at first scan (years)	72 ± 6
Age at second scan (years)	74 ± 6
Sex	20 females/8 males
Education (years)	12.34 ± 2.76
Handedness	26 R, 1L, 1A

*Abbreviations: R, right-handed; L, left-handed; A, ambidextrous*.

### Behavioral Measures

Participants underwent a battery of cognitive tests including 11 different measures which are briefly synthesized in Table 2 and reported in detail in other manuscripts (Mella et al., 2013, 2015; Fagot et al., 2018). Two of the measures, heterogeneity and amplitude of change, indicate an age-related change in cognition and the rest indicate baseline cognitive performance (i.e., measured at one timepoint). These measures are a composite score summarizing spatial and spatial-verbal working memory scores (Mat_span, MDA), crystallized abilities (MH), fluid intelligence (Raven), cognitive inhibition (Stroop), as well as mean performance and intraindividual variability in three reaction time tasks (a simple reaction time task, SRT) and a two-conditions complex processing speed task (CL6 and CL9). Intraindividual variability and mean performance have been shown to reflect different cognitive processes, intraindividual variability being linked to sustained attention abilities, while mean performance reflects the general level of processing speed. The composite scores of amplitude and heterogeneity of change in cognitive abilities at the individual level have been computed using individual analyses of variance of all tasks assessing simple reaction times, complex processing speed, and inhibition. This allowed assessing both the general amplitude of change and the heterogeneity of individual change across these tasks independently of the sample characteristics (see Mella et al., 2018 for more details). Briefly, heterogeneity of change refers to variations in the patterns of change across various cognitive measures. The homogenous decline is said to reflect a global decline in attentional resources or of mental resources, while strong heterogeneity of change may indicate a decline in one ability but stability or improvement in another (i.e., heterogenous pattern). Mean amplitude of change refers to both the direction and amplitude of the change in performance across the tasks considered. A smaller, negative change does not necessarily indicate decline but rather stability or less improvement. The higher the absolute value the stronger the change (explained in detail in Mella et al., 2018).

**Table 2:** Behavioral measures and corresponding cognitive function.

Behavioral task	Function	Mean (SD), range	Abbreviations
Single measures
Mill Hill Vocabulary Test	Crystalized intelligence	28.06 (4.52), 14–33	MH_MRI1
Raven Progressive Matrices 38	Fluid Intelligence	41.07(8.75), 23–56	RAVEN_MRI1
Matrices task as position recall	Verbal spatial working memory	2.48 (0.82), 0.35–4.45	MDA_MRI1
Matrices task as word-position recall	Spatial working memory	4.48 (1.12), 3–7	MAT_MRI1_SPAN
Reaction time task (IIV)	Reaction time	0.22 (0.05), 0.12–0.32	SRT_MRI1CV
Reaction time tasks (mean)	reaction time	325.11 (62.43), 241.01–497.81	SRT_MRI1M
Letters Comparison task (6L, IIV)	Processing speed	0.22 (0.05), 0.12–0.38	CL6_MRI1CV
Letters Comparison task (9L, IIV)	Processing speed	0.18 (0.06), 0.11–040	CL9_MRI1CV
Letters Comparison task (6L, mean)	Processing speed	2,969.51 (735.24), 2,090.50–4,822.61	CL6_MRI1M
Letters Comparison task (9L, mean)	Processing speed	4,438.97 (935.65), 2,975.93–6,961.79	CL9_MRI1M
Color Stroop Task	Resistance to interference	0.22 (0.10), 0.03–0.53	STROOP_MRI1
Composite measures
Heterogeneity of change in processing speed assessed in reaction time tasks of different complexity	Stability, improvement, or decline of performance across tasks	0.21 (0.01), 0.001–0.72	Heterogeneity
Amplitude of change (increase or decrease in performance)	Magnitude of the change	−0.044 (0,12), −0.34 to 0.21	Amplitude

*Note: All measures of tasks are given at baseline. IIV, intraindividual variability; IIV in reaction time and processing speed tasks are estimated with coefficients of variation*.

The 11 single measures of cognition, as well as the general amplitude and heterogeneity of change were correlated with change in SC and FC (described below) using Partial Least Square Correlation (PLSC; Mella et al., 2013, 2015; Fagot et al., 2018).

### MRI Acquisition and Preprocessing

Participants were scanned in a Siemens Trio 3T magnet. The following imaging sets were analyzed in the current study: resting-state functional MRI (fMRI), structural T1-weighted MR image (T1-w), and DWI sequences. Two sequences of 30 directions DWI were acquired (TR = 8,400 ms, TE = 88 ms, *b* value = 1,000 s/mm^2^, and voxel size 2.0 mm^3^). The two DWI acquisitions were concatenated using FSL 5.08^1^ (Smith et al., 2004) to increase the signal-to-noise ratio during the postprocessing. Then, a structural T1-w MR image was acquired (TE = 2.27 ms, TR = 1,900 ms, FOV = 256 mm, voxel size 1.0 mm^3^). Finally, the resting-state fMRI was obtained using an echo planar imaging acquisition (echo time, TE = 30 ms, time repetition, TR = 2,100 ms, flip angle = 80°, field of view, FOV = 205 mm, voxel size = 3.2 mm^3^, 140 volumes).

The diffusion data were corrected for eddy currents and movements using FSL (Andersson and Sotiropoulos, 2016). Structural T1-w MR images were preprocessed using Freesurfer version 6.0^2^ (Fischl, 2012) with a standard automated preprocessing pipeline (i.e., “recon-all” with the default set of parameters). Tissue-segmented images were obtained (i.e., white matter, gray matter, and cerebrospinal fluid), and used at the diffusion data analysis stage. For each participant, the T1-w image was registered to the diffusion data with cross-modal registration (i.e., rigid with six degrees of freedom) using a boundary-based cost function (Greve and Fischl, 2009). The diffusion data were further processed using MRtrix3^3^ (Tournier et al., 2019) following the following standard structural connectome construction steps. Briefly, to estimate white matter fiber orientation distributions the white matter response function was estimated and used to perform single-shell, single-tissue constrained spherical deconvolution (MRtrix command “dwi2 response Tournier”; Tournier et al., 2004, 2007, et al., 2013). Whole-brain tractography was generated using anatomical-constrained tractography (MRtrix command “tckgen”, 50 million streamlines, maximum tract length = 250, fractional anisotropy cutoff = 0.06; Smith et al., 2012). Spherical-deconvolution informed filtering of tractograms (SIFT2, command “tcksift2”) algorithm was used to estimate structural connection density (Smith et al., 2015; Tournier et al., 2015). Finally, the structural connectome was produced by mapping a multi-modal parcellation atlas with 360 regions (180 per hemisphere) described in detail in Glasser et al. (2017), to the streamlines obtained from SIFT2. A 360 × 360 connectivity matrix was created, representing the number of white matter reconstructed pathways for each pair of regions, normalized by the sum of the volumes of the two regions.

Resting-state fMRI was preprocessed using SPM8^4^ and functions of the data processing assistant for resting- state fMRI (Yan, 2010) and individual brain atlases using statistical parametric mapping (Alemán-Gómez, 2006) toolboxes. Standard preprocessing steps were followed: realignment of the functional scans, spatial smoothing with an isotropic Gaussian kernel of 5-mm full width at half maximum, co-registration of the T1-w image to the functional mean, and segmentation of the structural images (white matter, gray matter, cerebrospinal fluid). The average signal from cerebrospinal fluid and white matter then was regressed out from functional time courses, together with six motion parameters (translation and rotation along the three dimensions) and linear/quadratic trends. Next, Glasser’s multimodal parcellation atlas (the same used for the structural connectome) was resliced to functional resolution and applied to the fMRI data to estimate regional average time courses. For each participant, functional connectivity was calculated as Pearson correlation between each pair of time series. These were then Fischer z-transformed and stored in a 360 × 360 FC matrix.

For each subject, the change in SC and FC was calculated as the difference in connectivity values between time 2 and time 1, after taking the absolute values of the matrices for FC. The absolute change in connectivity at the individual level, reflecting therefore a change in connectivity strength (regardless of the sign, for FC), was then stored as final connectivity difference matrices.

Age and gender were regressed from the SC and Fischer’s z-transformed FC, and residuals were used for the PLSC analysis.

### Statistical Analysis

The behavioral measures (*n* = 13) were correlated (Pearson’s) with each other to better understand the relationships between them, resulting in a 13 × 13 cross-correlation matrix of correlation coefficients r. The p-values were corrected for multiple comparisons using a false discovery rate (FDR; Benjamini et al., 2006). MATLAB version 2019a^5^ was used for all statistical analyses.

PLSC for neuroimaging (McIntosh and Lobaugh, 2004; Krishnan et al., 2011) was used to assess the multivariate patterns of correlations between behavioral variables (i.e., cognition) and SC and FC measures. The performed analysis relates to that described in Zimmermann et al. (2018). Briefly, the upper triangle of the symmetric connectivity difference matrices (360 × 360 regions of interest) was vectorized for each of the subjects. Structural connections that were 0 at both timepoints, for 66% of subjects or more, were excluded from the analysis to avoid issues with resampling statistics (Zimmermann et al., 2018). Vectorized brain connectomes were then stacked across subjects. The behavioral variables were stored in a matrix of subjects^*^13 behavioral measures. Next, to start the PLSC, we computed a correlation matrix between the brain and behavioral matrices (i.e., a cross-correlation matrix between two data matrices). The cross-correlation matrix representing the brain-behavior correlation was submitted to singular value decomposition (SVD), which yields orthogonal latent variables (LVs) that capture the covariance between the variables from the behavioral and brain datasets (McIntosh and Lobaugh, 2004; Ziegler et al., 2013). The significance of the LVs was determined *via* permutation tests (1,000 iterations) of the singular values from the SVD, and the stability of the brain and behavior weights (also called saliences) was assessed using bootstrapping (500 bootstrap samples). For each significant LV, bootstrap ratios (BSR) for brain and behavior weights were calculated as the ratio of the weight over its estimated standard error. The stability of each connection in brain weights and behavioral weights was assessed based on its BSR: a positive high BSR >2.5 contributed positively and reliably to the brain-cognition correlations while a negative high BSR <−2.5 contributed negatively and reliably to the brain-cognition correlations. The positive and negative dimensions reflect distributions of connections that covary in a similar pattern to one another. Therefore, a BSR with a larger magnitude indicates that the connection with which it is associated has a large singular vector weight (i.e., contributes to the LV) and a small standard error (i.e., stable across participants).

To explore the contributions of each imaging modality to behavior, a matrix of the pairwise linear correlation coefficients (Pearson correlation coefficient) between each pair of regions was calculated. Next, each matrix was vectorized and correlated using Pearson correlation.

The PLSC analysis was conducted with the 13 cognitive measures (see Table 2) as behavioral variables and with SC and FC change as brain variables.

## Results

### Cognitive Measures

**Figure 1:**
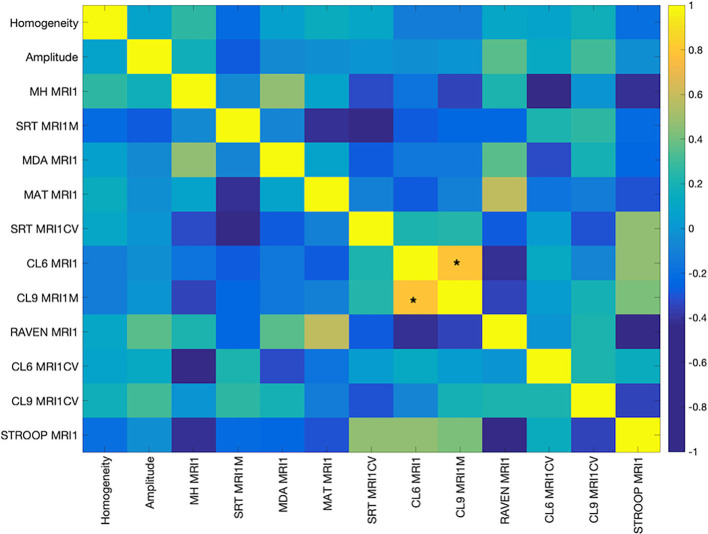
Pearson’s Correlation among the cognitive measures. The color bar indicates the strength of the correlation (r). Abbreviations: MH MR1, Mill Hill Vocabulary Test; SRT MRI1M, mean performance of Simple Reaction Time; MDA MRI1, Matrices Task with position recall; MAT MRI1, Matrices Task with word position recall; CL6 MRI1, mean performance on Letters Comparison Task with six letters; CL9 MRI1, mean performance on Letters Comparison task with nine letters; RAVEN MRI1, Raven Progressive Matrices 38; CL6 MRICV, Coefficient of variation of Letters Comparison task with six letters; CL9_MRI1CV, Coefficient of variation of letters comparison task with nine letters; Stroop MRI1, Color Stroop Task. *Indicates significance at *p* < 0.00001.

The cognitive measures were mostly weakly correlated with each other. Only measures CL9 MRIM1 and CL6MRI1M, both measures of processing speed involving mean performance on letters comparison task with nine letters and six letters, respectively, were positively and significantly correlated after FDR correction (0.83, *p* < 0.00001; Figure 1).

### Brain Connectivity and Behavioral Interpretation

The relationship between the change in FC and SC and cognitive function (resulting from the PLS analysis) was captured by one LV (17% of total covariance; singular value = 2,406.87; *p* = 0.0070), showing a significant contribution to the covariance. The LV revealed functional and structural correlations expressed across an array of behavioral measures, that optimally covaried with each other, and contributed negatively or positively to the FC and SC—cognition relationship. The contribution of the connections was expressed as BSRs, which indicates how robustly each connection contributed to the weighted pattern of the SC and FC—cognition matrix.

**Figure 2:**
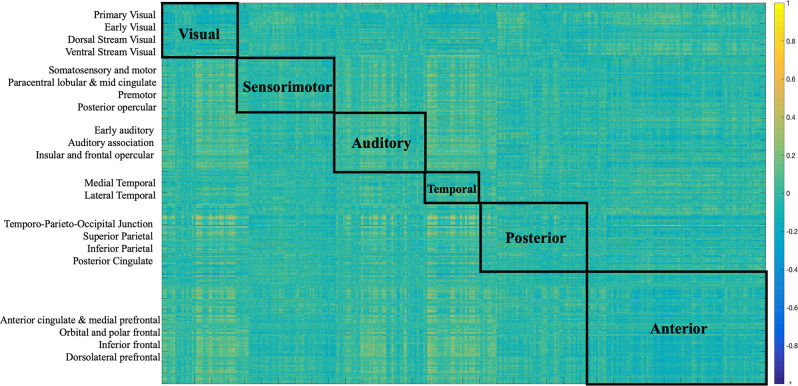
Correlation matrix between SC and FC modalities. The first 180 regions pertain to the left hemisphere and the next 180 (i.e., 181–360) to the right hemisphere (i.e., homologs). Their full description can be found in Table 1 in Supplementary Neuroanatomical Results in Glasser et al. (2017). The color bar refers to the strength of the correlations.

The two imaging modalities were overall weakly correlated (−0.072, *p* < 0.0001). The strength of correlations varied based on region as depicted in Figure 2. For example, regions in the posterior cortex (e.g., temporo-parieto-occipital junction, superior parietal, inferior parietal, posterior cingulate) were positively correlated with medial temporal and lateral temporal cortices. The anterior cortex and sensorimotor cortices largely showed negative correlations with the rest of the brain. Positive correlations indicate that both FC and SC change contribute in the same direction towards behavior, while negative correlations indicate that FC and SC change have opposite contributions.

**Figure 3:**
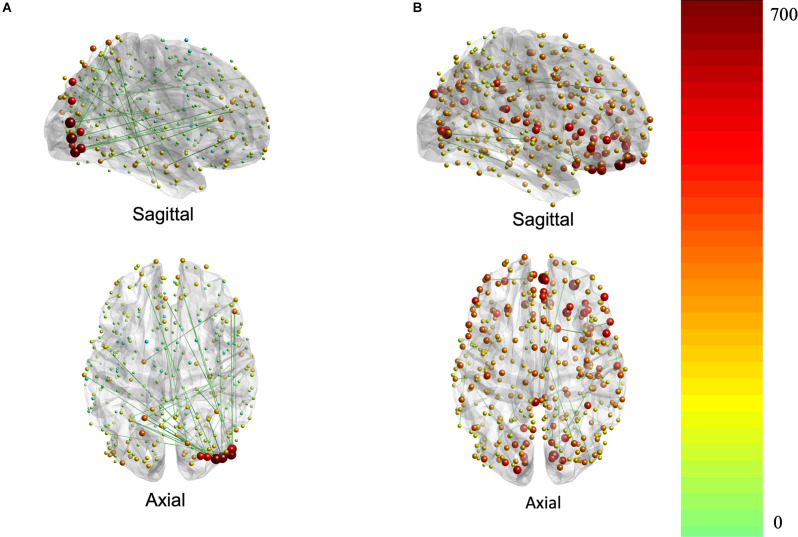
Brain plots for FC **(A)** and SC **(B)** brain saliences. Brain regions are represented by nodes whose size and color code are proportional to their node strength. The node strength essentially represents the degree to which each connection contributes to the covariance pattern of behavior (red is higher strength while green is lower strength).

The contributions to behavior can be better appreciated in Figure 3, which depicts the association of each region (i.e., node) to the brain-behavior relationship.

**Figure 4:**
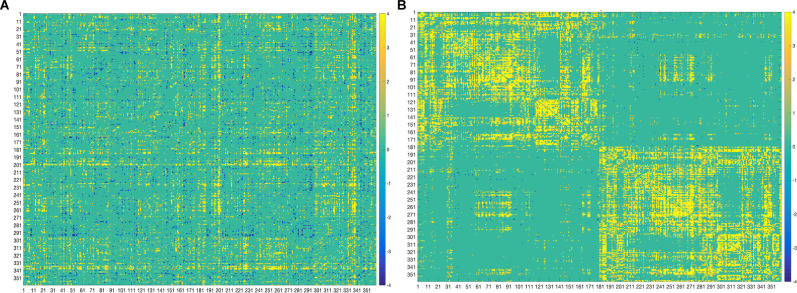
Bootstrap connectivity weights for FC **(A)** and SC **(B)** change. The first 180 regions pertain to the left hemisphere and the next 180 (i.e., 181–360) to the right hemisphere (i.e., homologs). Their full description can be found in Table 1 in Supplementary Neuroanatomical Results in Glasser et al., 2017. The color bar refers to the bootstrap ratios. Connections with positive bootstrap ratios are positively associated with the FC or SC—cognition correlation, and negative bootstrap ratios are negatively associated with the FC or SC—cognition correlation.

**Figure 5:**
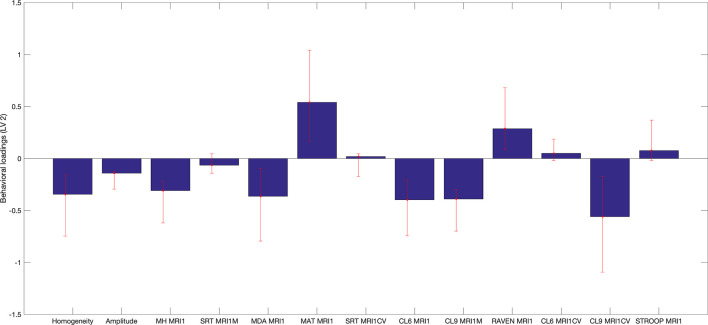
Behavioral saliences, with confidence intervals from bootstrap resampling. Error bars indicate bootstrapping 5th to 95th percentiles. Abbreviations: MRI1, at first MRI scan; MH MR1, Mill Hill Vocabulary Test; SRT MRI1M, mean performance of Simple Reaction Time; MDA MRI1, Matrices Task with position recall; MAT MRI1, Matrices Task with word position recall; CL6 MRI1, mean performance on Letters Comparison Task with six letters; CL9 MRI1, mean performance on Letters Comparison Task with nine letters; RAVEN MRI1, Raven Progressive Matrices 38; CL6 MRICV, Coefficient of variation of Letters Comparison Task with six letters; CL9_MRI1CV, Coefficient of variation of letters comparison Task with nine letters; Stroop MRI1, Color Stroop Task.

Specifically, several sets of functional and structural connections were found to have stable weights by bootstrapping (Figure 4). The behavioral weights corresponding to the significant LV are shown in Figure 5. In brief, Figure 5 indicates the behavioral pattern determined by the LV. PLS selects patterns of connections whose signal change covaries with the behavioral pattern across subjects identified by the LV. Those behaviors with higher BSR are said to modulate or contribute to a greater extent to the functional or structural connections than behaviors with lower BSR. The behavioral interpretation is presented in the following subsections (i.e., “Structural connections”, “Functional connections”) in conjunction with the brain weights.

Several sets of overlapping and non-overlapping functional and structural connections, specified in detail in the following subsections, were found to uniquely and independently contribute both negatively and positively to the association between change in brain connectivity and cognition in aging.

To facilitate interpretation of the data, in each hemisphere the 180 regions were separated into 22 larger partitions or “cortices” based on their topological proximity, common properties, based on architecture, task-fMRI profiles, and/or FC. Therefore, each of the 360 regions occupies one of the 22 cortices. The assignment is described in detail in Supplementary Neuroanatomical Results in Glasser et al. (2017).

#### Structural Connections

**Figure 6:**
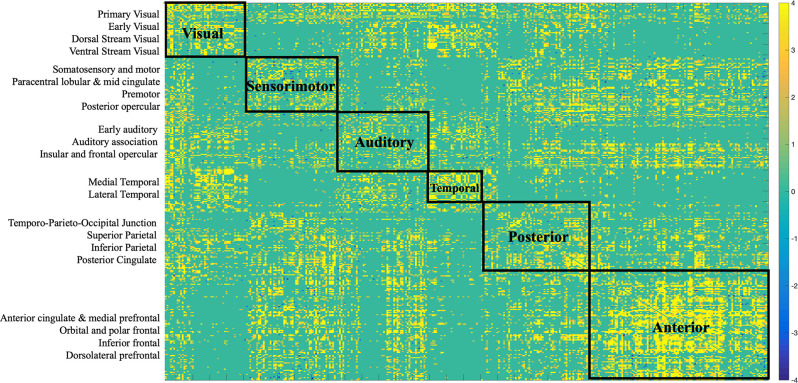
Bootstrap connectivity weights for SC change. Each of the 360 Glasser regions occupies one of the 22 cortices, which are listed on the y-axis. Each cortex was further labeled to a larger partition depicted on the matrix. The assignment is described in detail in Supplementary Neuroanatomical Results in Glasser et al. (2017).

Structural connections captured by the LV loaded mostly positively onto the brain-behavior relationship (Figures 3, 6). Some notable exceptions were several interhemispheric connections, mainly between the prefrontal cortex and several other regions (i.e., anterior cingulate, inferior frontal cortex, etc.), that loaded negatively. Specifically, interhemispheric structural connections between right dorsolateral prefrontal cortex and several other regions: left anterior cingulate and medial prefrontal cortex (BSR = −5.01), left inferior frontal cortex (BSR = −4.32), left premotor cortex (BSR = −5.01), dorsal visual stream (BSR = −5.01), left dorsolateral prefrontal cortex (BSR = −5.00), and intra-hemispheric structural connections between right insular and frontal opercular cortex—right lateral temporal cortex (BSR = −4.128), left superior parietal cortex (including intra-parietal sulcus) and left premotor (BSR = −4.16), and right dorsal stream visual cortex and left temporo-parieto-occipital-junction (BSR = −3.46), etc. Parieto-occipital-junction was defined as a strip of cortex bounded by auditory, lateral temporal, inferior parietal, and occipital (visual MT+ complexes) regions (Glasser et al., 2017). Therefore, decreased SC between the above-mentioned regions was positively correlated with cognitive functioning (i.e., measured by heterogeneity and amplitude of change), and baseline performance on crystallized intelligence, verbal-spatial memory, and processing speed. Decreased SC was negatively correlated with baseline performance on spatial working memory, and fluid intelligence.

#### Functional Connections

**Figure 7:**
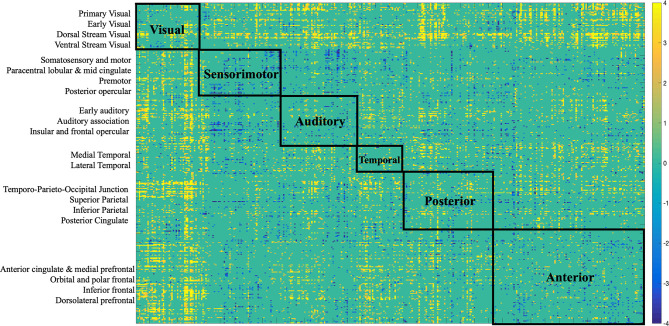
Bootstrap connectivity weights for FC change. Each of the 360 Glasser regions occupies one of the 22 cortices, which are listed on the y-axis. Each cortex was further labeled to a larger partition depicted on the matrix. The assignment is described in detail in Supplementary Neuroanatomical Results in Glasser et al. (2017).

There was variation in the contribution of FC connections to the brain-behavior relationship (Figures 3, 7). Some connections showed a strong, positive contribution including left premotor cortex and right early visual cortex (BSR = 8.57), left lateral temporal cortex and left temporo-parieto-occipital junction (BSR = 8.60), somatosensory and motor—MT+ complex and neighboring visual areas (BSR = 5.13). Thus, increased FC in these regions was correlated with inferior baseline performance on crystallized intelligence, verbal-spatial memory, and processing speed. Increased FC was positively correlated with higher scores in baseline spatial working memory, and fluid intelligence.

Others showed a strong negative contribution, including interhemispheric functional connections between right insular and frontal opercular cortex and several other regions: left paracentral lobular and mid cingulate cortex (BSR = −5.47), left anterior cingulate and medial prefrontal cortex (BSR = −3.32), left inferior parietal cortex (BSR = −5.72), posterior cingulate (BSR = −3.24), left somatosensory and motor cortex (BSR = −3.03), left superior parietal cortex and intra-parietal sulcus (BSR = −4.23). Also, intra-hemispheric functional connections between right between posterior opercular cortex and right posterior cingulate (BSR = −3.14), and right anterior cingulate and medial prefrontal cortex—insular and frontal opercular cortex (BSR = −4.23).

Therefore, decreased FC in these regions was positively correlated with cognitive functioning (i.e., measured by heterogeneity and amplitude of change), and baseline performance on crystallized intelligence, verbal-spatial memory, and processing speed. Decreased FC was negatively correlated with baseline spatial working memory and fluid intelligence.

## Discussion

In the current study, we characterized the mapping of age-related changes in FC and SC on 13 behavioral measures of cognitive function such as spatial working memory (MAT_MRI1_SPAN), crystallized intelligence (MH_MRI1), reaction time (SRT_MRI1M), and resistance to interference (i.e., STROOP_MRI1), in a sample of older adults.

We used PLSC, a multivariate method increasingly used for neuroimaging analysis (McIntosh and Lobaugh, 2004), to characterize brain-behavior relationships.

### Structural and Functional Connections Vary in Their Contribution to the Brain-Behavior Relationship in Aging

Several distinct sets of functional and structural connections were identified and assigned to 22 larger partitions (see “Materials and Methods” Section). This can be visualized in Figure 2. Areas that contribute to behavior in the same way (either both negatively or both positively) are depicted in yellow (i.e., positive correlations), while areas that have opposing contributions are in dark blue. Visual, auditory, and temporal cortices mostly present positive correlations between FC and SC contributions indicating that in these areas FC and SC change contribute in the same direction to behavior. Anterior cortices had largely a negative correlation with other cortices. For example, FC change (Figure 7) indicates negative loading onto behavior while SC change (Figure 6) indicates positive loading. This indicates that there is preservation of structural connections but decline in functional ones.

The majority of the structural connections loaded positively onto the latent variables identified by PLSC, indicating stability or increased connectivity. Given the relatively short time of 2.5 years between the scans, and that our study sample is of healthy older adults we did not expect an accelerated global decrease in structural connectivity. Although the majority of structural connections contributed strongly and positively to the brain-behavior relationship, there were some exceptions with some areas contributing strongly and negatively (see Figure 4). Notably, interhemispheric structural connections between the right dorsolateral prefrontal cortex and several other regions (frontal, sensory, motor) in the left hemisphere: anterior cingulate and medial prefrontal cortex, inferior frontal, premotor, dorsal visual stream, and dorsolateral prefrontal cortex. The dorsal visual stream contains higher visual areas implicated in perceiving where visual stimuli are located and in planning visually guided actions (Ungerleider and Mishkin, 1982; Goodale and Milner, 1992). Other negatively contributing regions were left intra-hemispheric fronto-parietal and temporo-parieto-occipital structural connections. Decreased SC in these regions was associated with the decline in cognitive functioning (i.e., measured by heterogeneity and amplitude of change), and inferior baseline performance on crystallized intelligence, verbal-spatial memory, and processing speed. Our results are congruent with other studies that indicate that normal aging involves decreasing myelination and reduced microstructural integrity of the white matter in the dorsolateral prefrontal cortex as well as in the corpus callosum (i.e., reduced interhemispheric connections), leading to reduced conduction of nerve fibers and cognitive decline (Peters and Sethares, 2002; Bowley et al., 2010; Kaller et al., 2015). Such changes are commonly seen in other prefrontal areas with aging and correlated with reduced cognitive ability especially involving processing speed, planning, and distractibility/inhibition (Eckert et al., 2010; Luebke et al., 2010; Kaller et al., 2015). Our measures of cognitive functioning, heterogeneity, and amplitude of change, were derived from tests assessing simple reaction times, complex processing speed, and inhibition. The findings of a decline in these measures correlating with decreased SC in prefrontal, motor, and sensory regions are in line with the existing literature. Studies indicate the decline in white matter fractional anisotropy is associated with lower processing speed, reflecting a regionally specific structural decline in attention-related regions in the prefrontal cortex, and a structural decline in the motor and sensory cortex, as these last are essential for performing processing speed tasks (Salthouse, 2000; Coffey et al., 2001; Charlton et al., 2006; Kennedy and Raz, 2009; Eckert et al., 2010).

There was variation in the contribution of functional connections to the brain-behavior relationship, which is in line with the existing literature, which indicates that age-related functional reorganization does not follow a global, whole-brain pattern rather is region-dependent (Goh et al., 2013; Cao et al., 2014; Zimmermann et al., 2016). Some connections showed a strong, positive contribution including somatosensory, motor, and temporo-occipital connections. Thus, preserved or increased FC in these regions was correlated with higher scores in baseline spatial working memory and fluid intelligence, decline in cognitive functioning, inferior baseline performance on crystallized intelligence, verbal-spatial memory, and processing speed. These observations are largely consistent with other studies reporting age-related increases in FC within motor cortices (Meier et al., 2012; Tomasi and Volkow, 2012), somato-motor connections (Betzel et al., 2014), and visual networks (Betzel et al., 2014; Geerligs et al., 2015).

Other functional connections showed a strong negative contribution to the brain-behavior relationship. Largely, these were composed of interhemispheric cingulo-opercular, and insular and opercular connections with parietal, paracentral lobule, medial prefrontal cortex, somatosensory, motor, anterior, mid, and posterior cingulate functional connections. To provide behavioral context to the above results, decreased FC in these regions was associated with the decline or less improvement in cognitive functioning (i.e., measured by heterogeneity and amplitude of change), inferior baseline performance on crystallized intelligence, verbal-spatial memory, processing speed, and higher scores in baseline spatial working memory, fluid intelligence.

Interestingly, decreased cingulo-opercular network (including the insula) FC has been reported to correlate with age-related reductions in visual processing speed (Ruiz-Rizzo et al., 2019), and so in our study, this may explain the lower scores on heterogeneity and amplitude of change since these were derived using processing speed tasks with visual stimuli. Some of the other functional connections mentioned (posterior cingulate, prefrontal, parietal cortex, etc.) are part of the default mode network (DMN; Alves et al., 2019), and lower DMN FC has been reported to be decreased in age-related decline in processed speed (Damoiseaux et al., 2008). We found that decreased FC between right insular and frontal opercular cortex and several other regions: left paracentral lobular and midcingulate cortex, left anterior cingulate and medial prefrontal cortex, left inferior parietal cortex, and posterior cingulate was associated with decreased cognitive functioning (i.e., measured by heterogeneity and amplitude of change), inferior baseline performance on crystallized intelligence, verbal-spatial memory, processing speed, and higher scores in baseline spatial working memory, fluid intelligence. Disruption of coordinated functional activity of key network regions such as the ones mentioned (posterior cingulate, prefrontal, parietal cortex), has been strongly linked to cognitive decline in advancing age without an overt disease (Andrews-Hanna et al., 2007). Some studies found that the relationship between decreased DMN and reduced processing speed is attenuated by controlling for whole-brain FC and white matter hyperintensities (Staffaroni et al., 2018) and reduced functional anisotropy between DMN areas (Andrews-Hanna et al., 2007). Therefore, there is a proposed vulnerability of these connections with cognitive aging. Similarly, reduced FC was reported for the insular and cingulated cortex with aging and cognitive decline (Onoda et al., 2012). Our study did not focus on within and between network analysis, however, that would be an interesting direction to explore. For example, the anticorrelation between the DMN and dorsal attention network is well-studied metric for cognitive functioning in aging. However, multi-modal longitudinal studies are limited in this area (Zhu et al., 2016).

Moreover, right intra-hemispheric functional connections involving cingulo-opercular, fronto-parietal, and prefrontal-opercular (including insula) connections, were also found to be negatively contributing to the brain-behavior relationship. In a comprehensive review by Robertson (2014), cognitive reserve (known to be protective in cognitive aging) is linked to the functional integrity of the frontoparietal connections in the right hemisphere, meaning that less FC in these regions may indicate a decline in cognitive functioning involving arousal, sustained attention, response to novelty, and awareness (Robertson, 2014; Haupt et al., 2019). Thereby, corroborating our results of decreased FC in these regions associated with a decline or less improvement in cognitive functioning.

### Future Directions and Limitations

A significant strength of our study is our longitudinal design, as only longitudinal studies can measure change. It allowed the investigation of individual brain-behavior developmental trajectories in aging, rather than just general tendencies in age differences, an important approach considering that aging is a heterogenous process. The within-subject approach minimizes cohort and period effects typical of cross-sectional designs and may help disentangle to what degree change in FC and SC ultimately drives aging-related changes in cognition, as well as investigate the behavioral relevance of FC and SC metrics.

Further longitudinal studies investigating age-related change in FC and SC while considering cognition are necessary to elucidate the underlying brain mechanisms behind cognitive decline in normal aging. It would be valuable to investigate the change in FC within and between brain networks (i.e., DMN, salience network, etc) and correlate it with white matter integrity and SC, with various brain parcellations, and across large longitudinal samples of healthy older adults and in different pathological states (i.e., Alzheimer’s).

Some weaknesses of our study are our relatively small sample size (*n* = 28) and the small number of cognitive measures that represent change (i.e., heterogeneity and amplitude of change). We also had only two timepoints at a relatively short interval (2.5 years), which may limit the generalizability of our study to the lifespan scale. Additional time points and a larger time frame would allow a more robust assessment of where on the trajectory of age-related change an individual’s brain is.

## Conclusions

In summary, we have shown that age-related changes in the brain-behavior relationship are supported by distinct positive and negative contributions from structural and functional connections distributed across the brain. From a behavioral perspective, we found that subjects with a decline in cognitive functioning (as derived from processing speed tasks), had decreased SC in fronto-parietal, prefrontal, and frontal connections, and decreased FC in cingulo-opercular, and DMN associated regions. Globally, we found a tendency for whole-brain SC preservation and mixed FC changes.

## Data Availability Statement

The raw data supporting the conclusions of this article will be made available by the authors, without undue reservation.

## Ethics Statement

The participants of the current study came from the longitudinal Geneva Aging Study. They all provided written informed consent, and approval was obtained from the ethics committee of the Faculty of Psychology and Educational Sciences of the University of Geneva and the Swiss Ethic Committee. The patients/participants provided their written informed consent to participate in this study.

## Author Contributions

All authors contributed to the design and implementation of the research, to the analysis of the results, and to the writing of the manuscripts. All authors contributed to the article and approved the submitted version.

## Conflict of Interest

The authors declare that the research was conducted in the absence of any commercial or financial relationships that could be construed as a potential conflict of interest.

## Publisher’s Note

All claims expressed in this article are solely those of the authors and do not necessarily represent those of their affiliated organizations, or those of the publisher, the editors and the reviewers. Any product that may be evaluated in this article, or claim that may be made by its manufacturer, is not guaranteed or endorsed by the publisher.

## Footnotes

^1^ http://www.fmrib.ox.ac.uk/fsl

^2^ http://surfer.nmr.mgh.harvard.edu/

^3^ http://www.mrtrix.org/

^4^ http://www.fil.ion.ucl.ac.uk/spm/

^5^ https://www.mathworks.com/products/matlab.html
